# How Can Imbalance in Oral Microbiota and Immune Response Lead to Dental Implant Problems?

**DOI:** 10.3390/ijms242417620

**Published:** 2023-12-18

**Authors:** Mansur Rahnama-Hezavah, Paulina Mertowska, Sebastian Mertowski, Julia Skiba, Karol Krawiec, Michał Łobacz, Ewelina Grywalska

**Affiliations:** 1 Chair and Department of Oral Surgery, Medical University of Lublin, 20-093 Lublin, Poland; mansur.rahnama-hezavah@umlub.pl (M.R.-H.); michal.lobacz@umlub.pl (M.Ł.); 2 Department of Experimental Immunology, Medical University of Lublin, 20-093 Lublin, Poland; sebastianmertowski@umlub.pl (S.M.); ewelina.grywalska@umlub.pl (E.G.); 3 Student Research Group of Experimental Immunology, Medical University of Lublin, 20-093 Lublin, Poland

**Keywords:** dysbiosis, microbiota, dental implants, osseointegration, immune system, dental implantology failures

## Abstract

Dental implantology is one of the most dynamically developing fields of dentistry, which, despite developing clinical knowledge and new technologies, is still associated with many complications that may lead to the loss of the implant or the development of the disease, including peri-implantitis. One of the reasons for this condition may be the fact that dental implants cannot yield a proper osseointegration process due to the development of oral microbiota dysbiosis and the accompanying inflammation caused by immunological imbalance. This study aims to present current knowledge as to the impact of oral microflora dysbiosis and deregulation of the immune system on the course of failures observed in dental implantology. Evidence points to a strong correlation between these biological disturbances and implant complications, often stemming from improper osseointegration, pathogenic biofilms on implants, as well as an exacerbated inflammatory response. Technological enhancements in implant design may mitigate pathogen colonization and inflammation, underscoring implant success rates.

## 1. Introduction

As clinical knowledge and dental technology continue to advance, dental implants are increasingly becoming the approach of choice for clinicians to effectively treat patients who have lost teeth for medical or mechanical reasons. More than 2.5 million implants are placed worldwide every year [1]. The use of dental implants is currently a treatment option, with a success rate of 97% after 10 years and 75% after 20 years after implant insertion [2,3]. Although such procedures are successful, their failure rate is 3.11% and is mainly related to the development of infections that occur through the proliferation of pathogenic bacteria in tooth gaps and pockets and the formation of a biofilm on the surface of implants. This results in the development of inflammation and excessive activation of the immune system, which translates into disturbances in the osseointegration process (resulting from the balance between the host’s immune cells and the bone biomaterials used), the development of hypersensitivity to dental materials used in implantology, as well as in periimplantitis [4,5,6]. Periimplantitis is considered one of the most difficult biological complications in dentistry, resulting primarily from poor osseointegration, the occurrence of chronic inflammation, and changes in the diversity of the oral microbiota, which, if left untreated, may result in complete loss of implants and the development of systemic diseases [7,8]. The composition of the oral microbiota includes over 700 types of microorganisms [9], dependent on, among other things, diet or environmental conditions.

Alterations in the oral microenvironment can lead to shifts in the biofilm’s microbial landscape, allowing specific bacterial strains to proliferate, increase their virulence, and become opportunistic pathogens. The oral cavity, which is a gateway to our internal body, deserves specific attention as the colonization of pathogens may lead not only to the loss of implants through the immune system but also to serious life-threatening diseases [10,11,12]. Dental implant complications can pose significant economic implications and impact patients perceptions of treatment [13].

As the prevalence of dental implants increases, the management of associated complications, such as oral microbiota dysbiosis and immune system disorders, continues to pose a significant challenge. The aim of this study was to present the most important findings regarding the impact of oral microbiota dysbiosis and the accompanying immune system dysfunctions in the context of dental implant failures.

## 2. Materials and Methods

### Search Strategy, Study Selection, and Data Extraction

The review of literature was conducted using the PubMed and Web of Science databases, with the search focusing on articles related to “dental implants”. Then, the available article search was narrowed down based on the time period from 2000 to 2023. Subsequent to the initial search, articles were further selected based on full-text availability and the inclusion of specific keywords such as “immune system” and “microbiota”. The articles that met these criteria were then reviewed by the authors for potential inclusion in the study. Duplicates were rejected at each stage of the analysis.

## 3. Diversity of Oral Microbiota and Its Interaction with the Immune System

**Figure 1:**
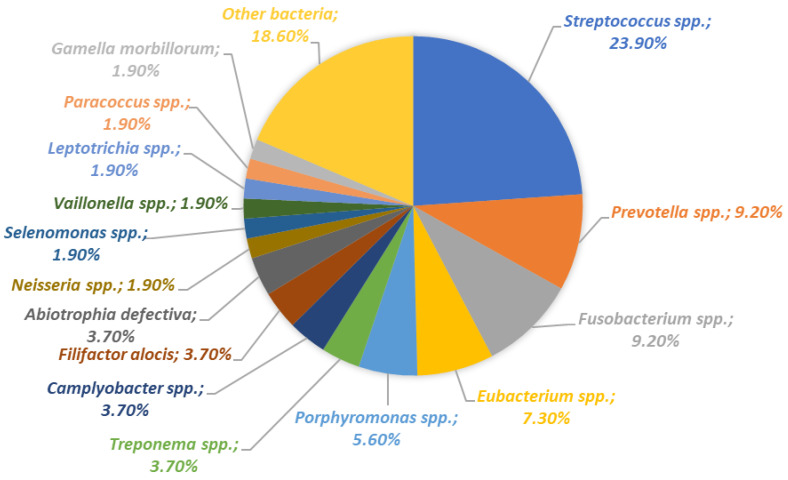
Normal composition of the oral microbiota (based on [14]).

The oral microflora includes a highly diverse and complex system of bacteria, microeukaryotes, archaea, and viruses. These microorganisms inhabit different niches in the oral cavity, such as the teeth, gingival sulcus, tongue, cheeks, and tonsils, and each provides a unique environment that influences microbial colonization and growth (Figure 1).

**Figure 2:**
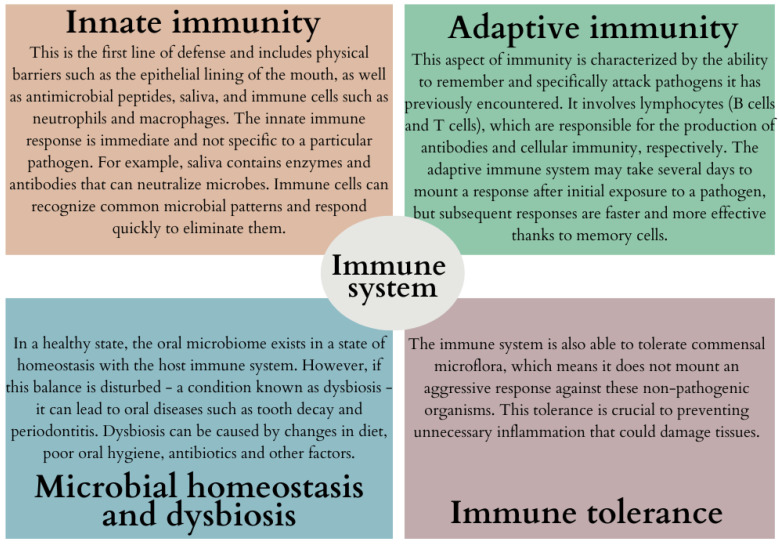
Examples of interactions between oral microflora and the immune system (based on [17,18,19,20]).

This diversity is not only inherent to oral health, but it also plays a key role in shaping local metabolic exchange due to the different microenvironments present in the oral cavity. The dynamic nature of these microbial communities is linked to the development of common dental diseases such as caries and periodontitis, highlighting the direct impact of oral microbes on human health [15]. The state of the host immune system and the diversity of bacteria within the oral microbiota are strongly correlated [16]. The interaction between the oral microbiota and the immune system appears to be a dynamic process involving both innate and adaptive immune mechanisms (Figure 2) [17,18,19,20].

**Figure 3:**
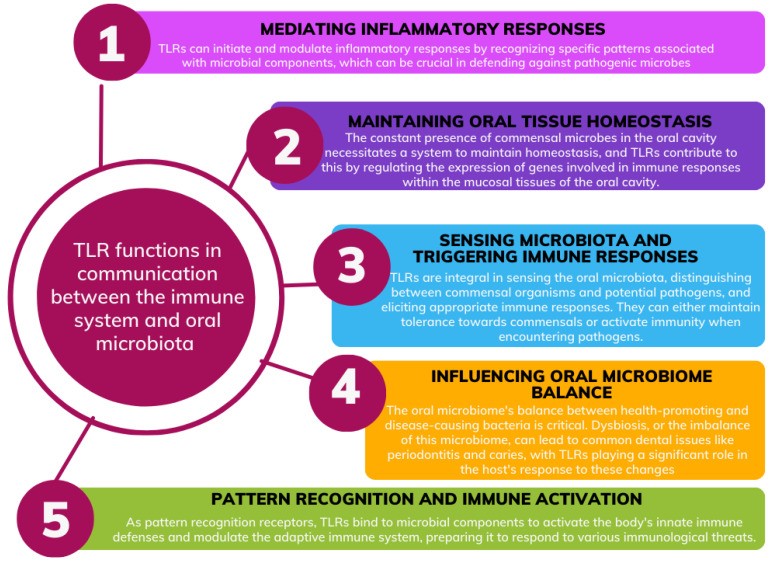
Examples of TLR (Toll-Like Receptors) functions in communication between the immune system and oral microbiota (based on [24,25,26,27,28]).

A balanced interplay between the microbiota and the immune system is essential for the proper initiation, modulation, and cessation of immune responses. Microbes interact with the immune system using pattern recognition receptors (PRRs), such as Toll-like receptors (TLRs), which are found on immune cells. These receptors can detect pattern-associated molecular patterns (PAMPs) and trigger appropriate immune responses [21,22,23]. TLRs are crucial for maintaining the delicate balance between commensal bacteria and the immune system in the oral cavity, playing a key role in maintaining oral cavity health. TLRs are expressed in oral epithelial cells and function as vital mediators of inflammatory pathways, acting as a bridge between innate and adaptive immunity. They serve several critical functions: mediating inflammatory responses, maintaining oral tissue homeostasis, sensing microbiota and triggering immune responses, influencing oral microbiome balance and immune activation (Figure 3) [24,25,26,27].

Many oral microorganisms establish biofilms, which are organized assemblies of microbes attached to surfaces and encased within a protective extracellular matrix. Biofilms are a natural part of the oral microbiome, but they might contribute to disease processes if pathogenic microorganisms dominate. Biofilms provide a stable environment for the complex community of microorganisms in the oral cavity. This protective matrix shields bacteria from environmental stress and allows for the maintenance of a balanced ecosystem [29,30,31]. Commensal bacteria within biofilms can contribute to oral health by outcompeting pathogenic bacteria for space and nutrients, thus preventing the disease.

The most commonly recognized biofilm in the oral cavity is dental plaque, a complex bacterial community that clings to the surfaces of teeth. Without regular removal, dental plaque can hasten the progression of tooth decay and periodontal conditions [32,33,34,35]. When the balance of the microbial community is disrupted, pathogenic bacteria might dominate the biofilm, resulting in dental caries as acid-producing bacteria thrive and periodontal disease as inflammatory pathways are triggered by the pathogenic biofilm [36,37]. Biofilms are more resistant to antimicrobial agents than planktonic bacteria. This resistance can complicate the treatment of oral infections and may require more aggressive and targeted therapies [38,39,40].

The varied composition of bacterial microflora across different regions of the oral cavity is apparent, influencing the efficacy and safety of various dental appliances utilized. This is particularly true in the context of dental implants and other dental materials, when the variety of bacteria and their specific adaptation to the oral environment underscore the need to tailor antibacterial methods to specific types of devices. Therefore, it should be considered that different areas of the oral cavity, due to their unique conditions, may favor the development of specific species of microorganisms, which requires an individual approach in terms of prevention and treatment [41,42,43].

Inflammation in the oral cavity, including periimplantitis, can be caused by various fungal species that form pathogenic biofilms and trigger inflammatory responses in the tissues surrounding dental implants. Studies have shown that strains such as Candida albicans are particularly capable of adhering to implant surfaces, which can lead to inflammatory conditions compromising implant success [44].

Oral biofilms can also pose systemic problems if pathogenic bacteria enter the bloodstream, potentially contributing to diseases elsewhere in the body. The control and management of biofilms is therefore a critical aspect of dental hygiene and overall oral health.

Biofilms can evade the immune system through physical barriers that prevent immune cells from penetrating and antimicrobial substances from being effective. The dense extracellular matrix can limit the diffusion of antibodies and phagocytic cells into the biofilm. Persistent biofilms in the oral cavity can culminate in chronic inflammation. The immune system’s continuous effort to combat biofilm bacteria can result in the overproduction of inflammatory cytokines, responsible for the tissue damage observed in periodontal disease [38,39,40,45,46]. Some biofilms can induce a tolerant immune response, which allows them to persist without causing overt inflammation. This can be a protective mechanism for maintaining a healthy balance of the oral microbiota [18,33,47]. Components of bacterial biofilms can sometimes mimic host tissues, potentially leading to autoimmune responses where the immune system attacks the body’s own cells. It is often more difficult for the immune system to remove microbes from biofilms, as the matrix can protect them from immune attack responses [29,48]. If the immune system successfully penetrates a biofilm, it can resolve the infection through mechanisms such as phagocytosis by neutrophils and macrophages and antibody-mediated neutralization [49,50,51]. The relationship between oral biofilms and the immune system is a key factor in the pathogenesis of oral diseases and in maintaining oral health. Understanding this interaction can help in designing strategies to prevent and treat biofilm-associated conditions.

This symbiotic relationship between the immune system and oral microbiota is crucial for preserving both oral and overall health, though it can lead to complexities owing to the immune system’s reliance on a varied microbial population [18,19]. Periodontal disease has been established as a contributing factor to systemic conditions, including cardiovascular disease and diabetes. The bacteria involved in periodontal disease can compromise the body’s immune defenses and prompt the release of inflammatory molecules such as IL-1β and TNF-α, which then enter the bloodstream from compromised periodontal tissue. This can lead to inflammation, potentially harming the vascular lining and contributing to the development of atherosclerotic plaque [52]. An elevated level of C-reactive protein, which is a response to oral bacterial infection, has a notable association with the onset of atherosclerotic vascular diseases [53].

Furthermore, oral bacteria are implicated in the incidence and progression of diabetes through the modulation of systemic immune stability [54]. Infections originating in the mouth can elicit a strong immune reaction in periodontal regions, resulting in a surge of inflammatory cytokines. A heightened reaction by mononuclear macrophages to these bacteria can trigger a localized release of high levels of IL-1β, IL-6, and TNF-α in cases of periodontitis [55]. The endotoxin from *P. gingivalis* can attach to CD14 molecules on macrophages, which activates the TLR2/4 signaling pathways. This activation process involves several intracellular interactions, culminating in the engagement of TNF receptor-related factors [56]. TNF plays a role in metabolizing lipids in fat cells, increasing circulating free fatty acids and decreasing insulin sensitivity, which can lead to insulin resistance and potentially worsen systemic inflammatory conditions, perpetuating a chronic imbalance in the inflammatory system.

The composition of the oral microbiome in individuals with liver cancer shows significant variance compared to healthy individuals [57]. Those with liver cancer have a more varied oral microbiome, with higher counts of bacteria such as *Bacillus*, *Leptotrichia*, *Actinomyces*, and *Campylobacter*, while bacteria such as *Haemophilus*, *Streptococcus*, and *Pseudomonas* are found in reduced numbers [57].

Oral pathogens may directly impact tumor development and progression via specific cytokines and biological pathways, or they may indirectly influence tumor dynamics by modulating the immune interactions between the tumor and host [58]. For instance, the prevalence of *P. gingivalis* is greater in areas affected by esophageal squamous cell carcinoma [59]. An imbalance in the oral microecology, often due to inadequate oral hygiene, may contribute to the buildup of carcinogenic agents and a persistent state of inflammation. Colorectal cancer, which ranks among the deadliest malignancies globally, has been found to have associations with an increased presence of Clostridium and *F. nucleatum* during its development [60]. Basically, the oral microflora and the immune system are in constant dialogue. Healthy microflora supports the functioning of the immune system, and an effective immune response prevents the overgrowth of potential pathogens, maintaining the balance necessary for both oral health and the health of the entire body. The interplay of oral microflora and the immune system is a finely tuned balance that is crucial to preventing disease and maintaining homeostasis in the body.

## 4. Complications in Dental Implantology: Understanding the Interplay between the Immune System and Oral Microbiota Dysbiosis

### 4.1. Types of Treatments and Composition of Implants Used in Dentistry

**Figure 4:**
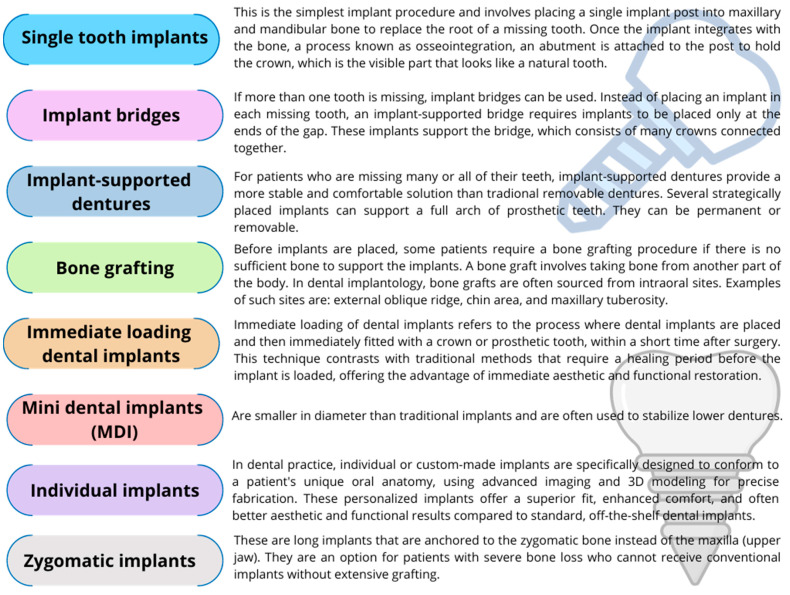
Examples of dental procedures used in dental implantology (based on [61,62,63,64,65,66,67,68]).

Dental implants are increasingly becoming the preferred treatment method for individuals who have lost teeth for various medical or mechanical reasons. In 2019, before the COVID-19 pandemic, over 3 million implants were placed in the United States [1]. Dental implantology includes several treatment procedures designed to replace missing teeth with artificial structures that resemble and function natural teeth (Figure 4).

Each of these treatments has its own specific indications, advantages, and considerations. The choice is based on the patient’s oral health, bone density, number of missing teeth, aesthetic requirements and the overall health. A thorough evaluation by a dental implant specialist is essential to determine the most appropriate treatment option for each individual patient.

**Figure 5:**
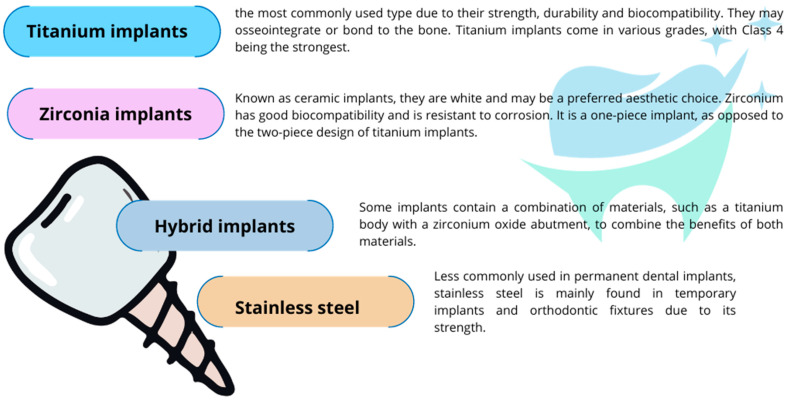
Types of implants used in dentistry (based on [70,71]).

A dental implant is a surgical component that is attached to the jawbone or skull to support prosthetic dental devices such as crowns, bridges, dentures, facial prostheses, or to serve as an orthodontic anchor [69]. These implants are fabricated from diverse materials, each chosen for its unique characteristics tailored to various clinical needs (Figure 5). 

**Figure 6:**
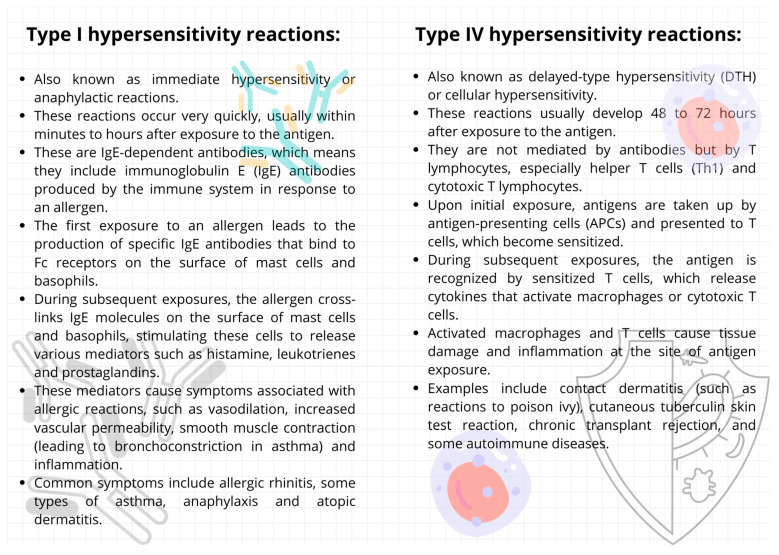
Comparison of the mechanisms of type I and IV hypersensitivity (based on [79,80]).

The choice of implant material depends on the patient’s needs, bone quality, aesthetic considerations, and potential allergies or sensitivity to different metals. Allergic reactions to metals used in dental implantology are a serious problem. The most common metals causing allergies in the context of dental implants are nickel and cobalt, the symptoms of which can include local reactions such as swelling, itching, and rash at the implant site, as well as systemic effects such as fatigue and weakness. Titanium, on the other hand, is considered highly biocompatible and hypoallergenic and is generally safe for individuals with metal allergies [72,73]. Regarding the overall safety of titanium, there is some evidence that a small percentage of the population may still experience allergic reactions to titanium dental implants. Research involving 1500 patients has indicated that, although rare, accounting for approximately 0.6% of cases, allergic reactions to titanium can occur in those with dental implants. Moreover, individuals with a history of metal allergies might have a higher propensity for titanium sensitivity, necessitating more studies to thoroughly comprehend the scope of these reactions [74]. Recently, there has been an increase in the incidence of oral allergies to metals used in dental materials, including titanium. Although allergic reactions to gold in dental prosthetics such as dentures have been acknowledged for a long time, titanium, frequently utilized in orthopedic fixtures and dental implants and considered biologically inactive, has been discovered to possibly trigger allergic responses, including both immediate (type I) and delayed (type IV) hypersensitivity [75,76]. Type I and type IV hypersensitivity reactions are distinct immune reactions that occur in the body and are classified based on the time of onset, the immunological mechanisms, and the cells involved, the detailed mechanisms of which are shown in Figure 6. Both types of hypersensitivity reactions can result from reactions to metals used in medical devices or implants.

Within dental implantology, type IV hypersensitivity reactions could be more prevalent. This occurs when the immune system responds to metal ions that are shed from the implant, potentially causing local tissue responses that might compromise the implant’s stability and integration [77,78].

**Figure 7:**
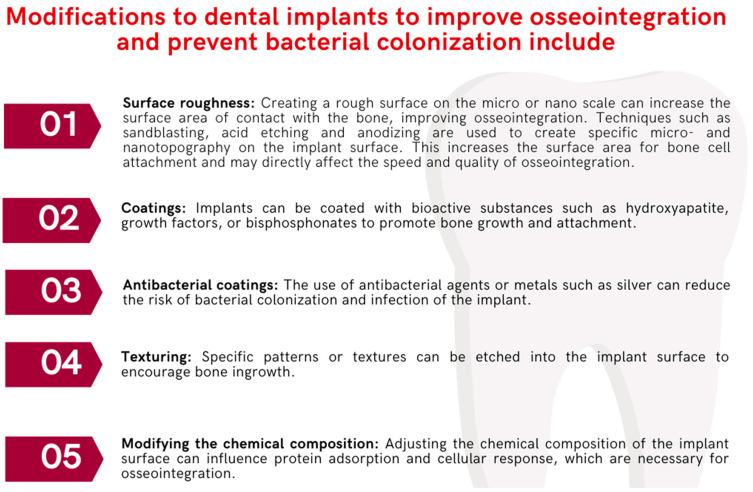
Types of implant modifications used in dentistry (based on [77,81]).

For patients with metal allergies, there are alternative materials, such as ceramics, that can be used to avoid potential allergic reactions. These materials are also an option for those individuals who care about the aesthetics of visible metal in their dental restorations. Moreover, frequently used dental implants are also subject to numerous modifications (Figure 7), which are aimed at:

- Enhanced osseointegration: To promote a faster and stronger bond between the implant and the jawbone, increasing implant stability and durability.
- Reduced healing time: Modifications can speed up the healing process, allowing for faster recovery and a shorter time to final reconstruction.
- Infection prevention: Modifications to incorporate antimicrobial properties are intended to reduce the risk of infections that may lead to implant failure.
- Improved biocompatibility: Surface modifications can increase the acceptance of the implant by surrounding tissues, reducing the risk of rejection.
- Improved aesthetics: Some modifications are intended to improve the appearance of implants, especially in visible areas of the mouth.
- Personalization: Customizing the implant to better fit the patient’s anatomy and specific clinical needs.

The primary objective of these enhancements is to secure the longevity and success of dental implants, which aids in enhancing oral health and increasing patient contentment. To augment the porosity, texture, and moisture affinity of the implant surface, as well as to add bioactive elements, a variety of nanocoating methods have been employed [82]. A coating of hydroxyapatite is applied to the surface of the implant to enhance osteoconductivity, thereby facilitating the process of osseointegration [83]. The surface of titanium nanoparticles is associated with an enhanced proliferation of osteoblasts and exhibits reduced bacterial adhesion and diminished biofilm maturation of pathogenic species such as *Porphyromonas gingivalis*, *Treponema denticola*, and *Tannerella forsythia*, after 30 days of exposure in comparison to other surface compositions [84]. The length and structure of dental implants are also a factor influencing the process of acceptance of the implant by the body. Implants with lengths exceeding 10 mm and designed with a square thread pattern tend to have higher success rates compared to those of shorter lengths or with designs lacking threads or featuring buttress threads [82]. Surface roughness is a critical factor impacting both osseointegration and biofilm formation, making it a primary focus for modifications to implant surfaces. Rough surfaces with a texture exceeding 2 microns are generally found to support better osseointegration compared to those that are smooth (less than 0.5 microns) or moderately rough (1–2 microns). Bacterial colonization of implants occurs within minutes after surgery. Determining the optimal surface roughness for implants remains a complex issue. To ensure a robust bond between bone and implant, a minimum surface roughness of 1–1.5 µm is necessary. However, surface roughness beyond 0.2 µm can lead to increased bacterial adhesion. Striking a balance between antimicrobial properties and favorable osteoconductive conditions is challenging; higher roughness levels may enhance bone integration but also correlate with greater bacterial adherence, potentially encouraging biofilm development [77].

### 4.2. The Critical Role of the Immune System in the Osseointegration Process of Dental Implants

**Figure 8:**
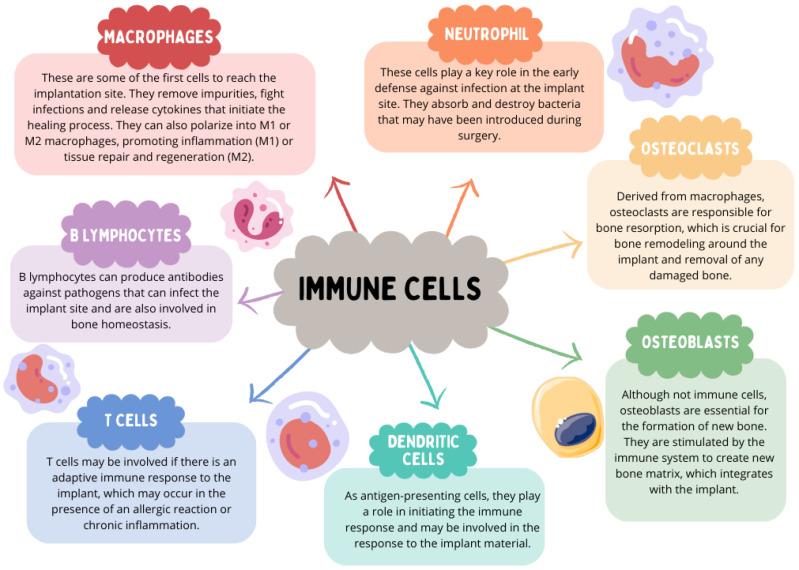
The role of immune system cells in the osseointegration process (based on [5,85,86]).

The immune system plays a pivotal role in the process of osseointegration in dental implants. Osseointegration is a long term equilibrium between host immune cells and bone biomaterials [4]. The role of the immune system in this process focuses mainly on 5 key aspects:

- Promotion of healing: After implantation, the immune system facilitates the healing of wounds at the site of implantation.
- Prevention of infections: Helps prevent potential infections that could interfere with the osseointegration process.
- Regulation of inflammation: Controlled inflammation is essential for osseointegration, but excessive inflammation can lead to implant failure.
- Supporting bone remodeling: Immune cells such as macrophages and osteoclasts are involved in bone remodeling, which is necessary for the integration of the implant with existing bone.
- The immune system plays a crucial role in maintaining homeostasis by regulating the balance between bone formation and resorption, a fundamental factor for implant stability. Numerous immune cells are engaged in these processes, including macrophages, neutrophils, osteoclasts, osteoblasts, dendritic cells, as well as T and B lymphocytes (Figure 8).

Macrophages hold a pivotal position in bone homeostasis and the integration of bone with biomaterials in the vicinity of dental implants. When foreign objects such as dental implants are introduced into tissues, macrophages fulfill a dual function by initiating either an inflammatory response (M1 macrophages) or an anti-inflammatory response (M2 macrophages) [87]. In patients with periimplantitis, M1 macrophages were found to be the predominant cell type [88]. A specialized subgroup of macrophages known as osteal macrophages plays a critical role in determining the outcome of implant osseointegration.

Osteal macrophages play a crucial role in bone by acting as immune surveillance cells. When a foreign biomaterial, such as a dental implant, is inserted into dental bone, there is often a rapid accumulation of macrophages on the implant surface [88]. During the process of dental implant osseointegration, classical M1 macrophages release a wide array of pro-inflammatory cytokines, including TNF-α, IL-1, IL-6, IL-12, MMP2, and MMP9. These cytokines are typically induced by factors such as IFN-γ + lipopolysaccharides or TNF-α. On the other hand, M2 macrophages are produced in response to IL-4 or IL-13 and secrete pro-regenerative cytokines, including PDGF-BB, TGB1, VEGF, IL-4, IL-10, and CCL18 [4].

Osseointegration, a critical process for the long term success of implants, is essential for implant stability and is considered a prerequisite for loading and the extended clinical success of dental implants in osseous tissue [89]. The implant-tissue interface is a highly dynamic region [90]. The immune system’s response and inflammation require active biochemical processes to restore homeostasis, ultimately facilitating implant osseointegration [91]. Poor osseointegration and persistent inflammation are the two primary factors underlying the development of peri-implantitis [92]. Initial damage to peri-implant tissues triggers an inflammatory response mediated by various innate immune cells, including macrophages, dendritic cells, mast cells, and neutrophils. Macrophages, in particular, play a central role in the initial immune response to implants. When the body is exposed to the implant material, the primary phagocytes that are activated in the early inflammatory stage are macrophages [87].

The presence of M2 macrophages in peri-implant tissue is linked to decreased inflammation, enhanced wound healing, and ultimately, the successful integration of implants into the bone [93]. In summary, macrophages may play a dual role in directing implant failure or success. Indeed, while M2 macrophages promote osseointegration and facilitate efficient wound healing, M1 macrophages can exacerbate the inflammatory process and accelerate osteolysis, ultimately contributing to the failure of dental implants.

Dendritic cells (DCs) are pivotal in initiating the immune response by presenting antigens to T cells. Langerhans cells (LC), a type of DC, are present in stratified epithelial layers such as the skin’s epidermis and the oral mucosa’s epithelium [94]. These cells modulate the oral mucosa’s immune environment and help protect it during infections [95]. Although there’s an increase in LC precursors within the peri-implant epithelium, the actual presence of mature LCs decreases. This could indicate that implants may interfere with the normal development and regulatory function of immune responses in the surrounding tissues [96]. Notably, the quantity of LCs, marked by CD1a, is higher in the epithelium and lamina propria of non-implanted healthy mucosa compared to that around implants. This reduction in LCs could lead to diminished immune responses in peri-implant tissues [97].

Neutrophils are critical initial responders in peri-implant tissues, engaging in the inflammatory response by producing cytokines and chemokines and forming structures known as neutrophil extracellular traps (NETs) [94]. The presence of fewer pro-inflammatory macrophages in response to NETs has been associated with successful bone integration [98].

Mast cells (MCs), originating from hematopoietic stem cells in the bone marrow, travel through the bloodstream and reside in various tissues. They have a dual role, promoting defense and bone integration and aiding in tissue repair if activated transiently. However, persistent and excessive activation can lead to severe osseointegration failure or peri-implantitis, causing extensive tissue damage [99,100]. The activity of innate immune cells is crucial in the body’s response to dental implants, and monitoring their numbers may enhance implant longevity and help prevent implant rejection.

### 4.3. Types of Complications Observed in Dental Implantology and Etiology of Dental Failures

The use of dental implants is currently a reliable method for treating edentulism, with a success rate of 97% after 10 years and 75% after 20 years [2]. However, as with any procedure, complications can arise that may result in implant loss. Implant failure can be categorized into early (<3 months) and late (>3 months) stages [2]. Early implant failure can be caused by factors such as excessive heating of the bone during drilling, over-preparation of the surgical site, or low-density bone that hinders the initial stability of the implant. Conversely, late implant failures are typically associated with infections, with periimplantitis being the most common [101]. The risks associated with dental implant failure are much debated among clinicians in order to avoid implant-related complications. With significant advancements in materials science and surgical techniques, there is a growing focus on patient-related factors as potential risk factors for dental implant failure [102]. Patients who have been exposed to radiation before or after implantation, as well as those with severe diabetes or a history of smoking, all face a significantly elevated risk of dental implant failure. These conditions can compromise implant survival by increasing a patient’s vulnerability to other diseases or interfering with tissue healing. Notably, smokers or individuals who have undergone radiation therapy before or after implantation may experience approximately a 35% or 70% higher risk of dental implant failure, respectively, compared to nonsmokers or those without a history of radiation therapy [103]. Clinicians should exercise caution throughout the entire dental implant treatment process—from initial examination through treatment planning, surgery, and denture selection—to minimize the risk of late dental implant failure. Smoking threatens both bone and wound healing processes [104].

Bruxism is a significant risk factor that can jeopardize the longevity of dental implants [105]. It is linked with high and irregular occlusal pressures that may lead to a spectrum of issues during implant therapy, including biological and mechanical complications such as implant surrounding bone loss, damage to dentures, screw loosening, and breakage of implant components.

The patient’s oral history, especially a history of periodontitis, stands out as a critical risk factor for the eventual failure of dental implants. Periodontitis, a leading cause of tooth loss, necessitates the use of implants for oral rehabilitation. Previous periodontitis is often seen as an indicator of potential peri-implantitis, which can result in the delayed failure of the implant [104]. The likelihood of transferring periodontal pathogens from the natural teeth to the implant site may account for this risk. Additionally, implants are at increased risk of failure when placed in areas of low bone density. Specifically, type IV bone quality, characterized by thin cortical layers and low trabecular density, markedly raises the probability of both early and delayed implant failures [106]. Such poor bone quality is closely linked to a lack of initial implant stability. Moreover, implants with a machined surface tend to have higher failure rates. In contrast, conventional threaded implants that are at least 10 mm long with an SLA (Sand-blasted, Large-grit, Acid-etched) surface tend to perform better in patients with sufficient bone mass. Conversely, shorter implants, no more than 7 mm, utilizing SPS (Spark Plasma Sintering) technology, have shown improved success in cases where significant bone loss has occurred [107]. Periimplantitis and peri-mucosal inflammation are generally observed in patients with unclosed crown margins, loose crown-retained screws, loose retaining screws, and broken retaining screws. When the denture is not properly positioned, it causes difficulties with proper hygiene, which leads to the formation of biofilm and future peri-implantitis. Periimplantitis and perimucositis had a lower incidence when using the Straumann system, whereas the Osstem system showed a higher incidence of periimplantitis. Exposure to cigarette smoke alters the composition of the bone matrix and hinders bone mineralization, resulting in increased bone fragility. Smoking reduces the thickness of bone trabeculae, leading to a decrease in the mineralizing surface and the rate of mineral deposition. All this consequently leads to a lower rate of bone formation and a longer mineralization time. The higher the dose and the longer the smoking time, the greater the effect on bone mineral density. Smokers present higher levels of free radicals and higher levels of oxidative stress biomarkers [108] than non-smokers, which may play an indirect role in activating bone proresorption pathways by influencing osteoclast differentiation and activity [109]. Smoking can also impact the RANKL-RANK-OPG pathway, which comprises a series of biochemical processes that regulate osteoclast proliferation and activity. This disruption of the pathway can ultimately hinder the bone healing process.

### 4.4. The Role of Oral Microbiota and the Immune System in the Course of Peri-Implantitis

**Figure 9:**
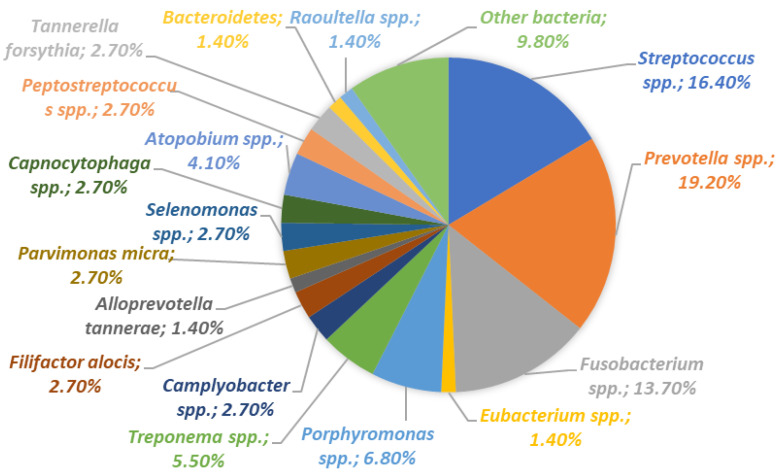
Dysbiosis of oral microflora (based on [14]).

Periimplantitis is a destructive and inflammatory disease that affects both the hard and soft tissues surrounding osseointegrated implants. It is characterized by progressive damage to the alveolar bone [110]. Mucositis occurs in approximately 80% of patients with dental implants. However, the incidence of periimplantitis ranges from 28% to 56% among patients and from 12% to 43% among implants [111]. Failure of dental implant function is classified as either early or late [112]. The occurrence of periimplantitis in a patient after implant insertion is classified as a late failure. In cases of late implant failures, the previously integrated implant’s normal function is disrupted due to chronic infection of the peri-implant tissues [112]. A characteristic clinical symptom of periimplantitis is an increase in the depth of the pocket, often accompanied by bleeding and, in some cases, suppuration [113]. In the first stage of the disease, the soft tissues around the implant become inflamed. This stage is called mucositis. Next, the extent of inflammation increases and exceeds the gum-bone boundary. The disease progresses into peri-implantitis. At this point, bone destruction starts. The longer the process lasts, the greater the bone loss. The oral microbiota is remarkably diverse, comprising over 700 distinct species, with an individual typically hosting at least 100 different types within their mouth [114]. Alterations in the oral environment (as depicted in Figure 9) can lead to a shift in the biofilm’s microbial makeup, potentially enabling certain bacterial species to proliferate, increase their pathogenic potential, and become opportunistic. The onset of periimplantitis is marked by the emergence of Gram-negative, motile, and anaerobic bacteria, which are also known to be prevalent in periodontitis [115]. Various microbes have been pinpointed in cases of periimplantitis, including *Staphylococcus aureus*, *Staphylococcus epidermidis*, *Enterobacter aerogenes*, *Enterobacter cloacae*, *Escherichia coli*, *Helicobacter pylori*, *Pseudomonas species*, and *Candida species* [116]. Notably, *S. aureus* is recognized for its adaptability and is often highlighted in orthopedic discussions as a primary cause of implant-related infections and subsequent bone infections or osteomyelitis [117]. Tissue from periimplantitis-affected areas has been found to express elevated levels of inflammatory agents such as the cytokines IL-6, IL-8, and TNF-α when compared to non-affected tissues [118]. Periimplant fissure fluid (PICF) represents the inflammatory discharge found in the narrow space between an implant and the surrounding mucosa, known as the peri-implant sulcus or fissure. Higher concentrations of TNF-α, IL-17, and IL-1β are present in PICF collected from sites affected by peri-implantitis. Damage to peri-implant tissues triggers an inflammatory response involving the activation of innate immune cells such as macrophages, dendritic cells, mast cells, and neutrophils. Neutrophils play a role in promoting the release of pro-inflammatory cytokines, including IL-1 and TNF-α. These cytokines, in turn, contribute to the osteolytic and inflammatory tissue damage characteristic of periimplantitis [119].

Periimplantitis is associated with a diverse range of bacterial species, including *Tannerela forsythia*, *Porphiromonas gingivalis*, *Aggregatibacter actinomycetemcomitans*, *Prevotella intermedia*, and *S. salivarius* [120]. While there are etiological similarities between the biofilm found in periodontitis and periimplantitis, the predominant bacteria in periimplantitis, as compared to healthy implants, are *Fusobacterium* spp. and *Treponema* spp. [121]. Over time, the analysis reveals an increase in the Firmicutes phylum during the maturation of the peri-implant plaque. After the establishment of periimplantitis, a decrease in the detection of *Neisseria* spp. and *Porphyromonas* spp. is observed [122]. Inflammation around a dental implant is considered one of the most difficult biological complications in dentistry because, if left untreated, it can progress and cause complete loss of implants. Treatment of periimplantitis requires extensive resources. Prevention of the disease is therefore a high priority in everyday dental practice to minimize the occurrence and severity of the problem.

## 5. Methods of Preventing Failures in Dental Implantology

### 5.1. Enhancing Dental Implant Safety: Strategies for Minimizing Bacterial Adhesion and Infection Risk through Material Modifications

Modifications to the physicochemical properties of dental implants play a role in diminishing the attachment of microorganisms to the implant surface. This has a beneficial impact on lowering the risk of initiating the periimplantitis process, although it cannot entirely eradicate this issue. The characteristics of dental materials influence not just the quantity of microorganisms but also their composition and how strongly they adhere to the material surface. Titanium has been the leading biomaterial used in dental implantology therapy for many years. However, despite titanium being a promising biomaterial that fulfills the requirements of modern dentistry in terms of suitable mechanical and biological properties, its antimicrobial capabilities fall short of preventing microbial colonization [123]. Hydrogels are employed to modify dental materials with the aim of enhancing their antibacterial properties. These hydrogels are hydrated polymers known for their remarkable therapeutic versatility, specifically designed for human use. These biomaterials create a robust network of either natural or synthetic molecules capable of encapsulating therapeutic agents within their internal structure. The biomaterials utilized in hydrogel production encompass polysaccharides (such as dextran and chitosan) and proteins (including gelatin and fibrin) [124]. When it comes to synthetic biomaterials, we include: polyvinyl alcohol [125] and polyethylene glycol [126] are widely used examples of hydrogel-forming polymers. Hydrogels have been shown to work effectively as a coating on titanium implants without causing an inflammatory reaction. The hydrogel may contain an antibiotic that will provide local protection of the implants against bacteria without disturbing the position of the bone or causing a local or systemic inflammatory reaction [127].

The versatility of hydrogels positions them as a compelling option for preventing implant-related infections. To enhance the efficacy of mechanical cleaning in peri-implant pockets, drugs are incorporated into the Layer-by-Layer (LbL) system at the implant insertion sites [128]. Ultraviolet (UV) radiation has also been employed in the modification of dental materials. Implant surfaces exposed to UV radiation exhibit increased bioactivity and enhanced osseointegration potential. This transformation is attributed to alterations in the surface layer of titanium dioxide. Furthermore, UV radiation enhances osteoconductivity by facilitating interactions between cells and proteins with the implant surface. It additionally reduces surface hydrocarbons, improves wettability, enhances protein adsorption, boosts cellular adhesion to titanium surfaces, and restores bioactivity that diminishes due to age-related degradation [129]. Tantalum may pose significant competition on the dental materials market for titanium currently used in dental implants. In many ways, tantalum is superior to titanium, stainless steel, and other metals. Currently, tantalum is widely used in the field of orthopedics. Among other things, tantalum has greater corrosion resistance and has therefore been successfully used as an implant material in orthopedic surgery to improve angiogenesis and wound healing [130]. Implants made of tantalum are more porous than titanium implants, they are also more flexible and their surface is rough. The use of tantalum in implants has a positive effect on the osseointegration process, which is crucial for implant success. Implants made of tantalum are more porous, which facilitates faster fusion of the implant with the bone. Furthermore, tantalum coatings have demonstrated the ability to enhance the proliferation of gingival cells [131], allowing them to firmly adhere to periodontal tissue, thereby decreasing the infection risk. Notably, tantalum exhibits superior antimicrobial properties compared to titanium. Pure tantalum surfaces exhibited lower adhesion rates for *Staphylococcus aureus* and *Staphylococcus epidermidis*, implying that tantalum may possess more effective antibacterial characteristics than titanium [132]. Ongoing research is exploring the utilization of tantalum as a biomaterial in the development of dental implants. However, due to its promising osseointegrative and antibacterial properties, this metal may become a potentially leading dental biomaterial [133].

### 5.2. Use of Probiotic Therapy

Current rates of antibiotic resistance related to microbiota dysbiosis caused by the widespread use of antibiotics and antiseptics have led to probiotics being suggested as a treatment option for peri-implantitis. Probiotics are defined as live microorganisms that, when administered in appropriate amounts, provide health-promoting benefits to the body.

Probiotics are deemed to be safe and beneficial due to their capacity to lessen the immunogenic properties of microbiota [134]. They accomplish this by fostering a more favorable microbial balance within the host, effectively suppressing harmful pathogens. Probiotics engage in a competitive interaction with oral microbes and disease-causing organisms for binding sites and essential growth elements, thus safeguarding dental health. By adhering to the oral cavity, these beneficial bacteria form clusters that block harmful bacteria from attaching by secreting antimicrobial substances such as acids, bacteriocins, and peroxides. Consequently, probiotics can suppress the proliferation of cariogenic and periodontal pathogens, bolster immune defenses against these harmful agents, and avert the breakdown and inflammation of oral tissues [135].

Furthermore, probiotics are instrumental in deterring the formation of dental plaque by acidifying saliva and synthesizing antioxidants, which neutralize free radicals necessary for plaque mineralization. This acidic environment thwarts the development of plaque by pathogenic bacteria. Numerous research findings indicate the efficacy of probiotics in managing dental plaque, gingivitis, and periodontitis and substantially diminishing the number of periodontal pathogens [136]. Maintaining a balanced oral microbiome is key to immune equilibrium and, as a result, diminishes the secretion of pro-inflammatory cytokines. *Lactobacillus* species have been used for years to balance the gut and vaginal microbiota and are now also proposed to balance the oral microbiota [134]. Bacteria of the *Lactobacillus* species are able to inhibit pathogenic bacteria such as *S. mutans*, *A. actinomycetemcomitans*, *P. gingivalis,* and *P. intermedia*. The strongest antimicrobial activity was associated with *L. paracasei*, *L. plantarum*, *L. rhamnosus*, and *L. salivarius* [137]. A probiotic based on *L. reuter* showed a slight reduction in the incidence of peri-implantitis.

Nonetheless, it exhibits anti-inflammatory properties and mitigates mucous membrane inflammation [138]. Clinically, it has proven effective in reducing pocket depth during peri-inflammatory treatment, although it may not always reach baseline levels [119]. Another approach involves the use of an *S. salivarius*-based probiotic to combat implant biofilm formation [139]. This probiotic functions through the interaction of a bacteriocin produced by *S. salivarius*, which inhibits quorum sensing signals and reduces the production of *S. intermedius* biofilm on the surface of titanium dental implants. While this probiotic shows promise for non-surgical therapy aimed at preventing implant diseases associated with bacterial biofilm formation, it has proven ineffective in treating peri-implantation diseases caused by the pathogen *C. albicans* [139].

### 5.3. The Importance of Selected Nutrients in the Osseointegration Process

The dietary habits of patients play a pivotal role in preventing or treating various diseases, including the osseointegration process of dental implants. Several micronutrients, notably calcium, fluoride, magnesium, potassium, vitamin B6, vitamin D, and zinc, have demonstrated their positive impact on bone health, lowering the risk of fractures [140]. Conversely, diets rich in fats, carbohydrates, cholesterol, and low in calcium have adverse effects on the jawbone [141]. Specific dietary patterns and micronutrients can play a key role in the various phases of osseointegration of dental implants. The information you’ve provided highlights a significant dietary concern according to National Health and Nutrition Examination Survey (NHANES) data—over a quarter of the US population is not consuming adequate amounts of essential nutrients such as vitamins A, C, D, E, and minerals such as calcium, magnesium, and potassium. This trend suggests that the typical Western diet may not be meeting the necessary micronutrient requirements for optimal health. It’s an important public health issue that may require dietary adjustments or policies to address nutritional deficiencies. Micronutrient deficiencies affect approximately two billion people worldwide [142]. The increase in the consumption of processed food contributes to increased micronutrient deficiencies in the diet, which has health consequences for the entire body. Vitamin C is also a vitamin considered to have a positive effect on the osseointegration process. It can inhibit the effects of oxidative stress by promoting bone resorption and, consequently, inhibiting the reduction of bone strength [143]. Osteoporosis, being an extremely common disease in the elderly, is a disease that disturbs the integrity of the implant. Osteoporosis considerably affects bone deterioration near dental implants [144]. Administering vitamin D helps mitigate this bone attrition, showing overall positive outcomes for the bone growth surrounding the implant. A widespread issue, vitamin D scarcity, is noted among the population at large. It plays a crucial role in bone mineralization and is also vital for immune function and managing inflammation. It does this by boosting anti-inflammatory agents and reducing inflammation-promoting agents [145]. Additionally, vitamin D’s influence on bone integration may extend to healing of the soft tissue and creating tighter seals at the implant’s edge, potentially enhancing resistance to bacterial contamination in the surrounding sulcus. There is an effect of active vitamin D3 on the expression of pro-inflammatory and anti-inflammatory cytokines (IL-6, IL-8, IL-10, and IL-12) in human gingival fibroblasts and human periodontal ligament cells. Vitamin D supplementation appears to improve osseointegration in people with systemic diseases such as vitamin D deficiency, diabetes, osteoporosis, and chronic kidney disease. Diabetes is classified as a disease that hinders the osteointegration of implants due to the impaired wound healing process in people diagnosed with type I and II diabetes [146]. Combination therapy with insulin and vitamin D showed the best effect on osseointegration, bone volume, mean trabecular thickness, mean trabecular number, total density, mean trabecular separation, push-out force, shear force, BIC and bone area ratio [147]. Magnesium deficiency led to lower cortical bone thickness, lower implant removal torque values, and lower bone mineral density (BMD) [148].

## 6. Discussion

The manuscript presents a nuanced exploration of the interactions between oral microbiota, immune responses, and their impacts on dental implant success, emphasizing the need for an in-depth understanding of these interactions for effective prediction, prevention, and management of dental implant complications. A key aspect discussed is the role of dysbiosis in hindering proper osseointegration, a fundamental process for implant success. Dysbiosis can lead to the formation of pathogenic biofilms on implants, disrupting the osseointegration process and contributing to implant failures [149,150,151].

Furthermore, the paper emphasizes the importance of technological advancements in dental implant designs, particularly those that reduce pathogen colonization and inflammation. Innovations in implant surface materials and coatings are crucial in addressing challenges posed by oral microbiota imbalances or immune response. The significant economic and clinical implications of dental implant complications are also touched upon. Understanding the etiological factors behind implant failures can help in developing more effective prevention and treatment strategies, thereby reducing the economic burden and enhancing patient outcomes [152].

The study opens pathways for future research, especially in personalized dental implantology. Tailoring implant treatment based on individual microbiota profiles and immune responses could become a frontier in implant dentistry. Additionally, the findings of this study hold significant implications for clinical practice, guiding dental professionals in managing implant treatments more effectively [153].

While the manuscript provides valuable insights, it could benefit from a more structured discussion section that directly links the research findings with existing literature and clinical practices. Including a section that addresses the limitations of the study and proposes future research directions would enhance the manuscript’s contribution to the field. In conclusion, by offering a detailed analysis of factors critical to dental implant failures, the manuscript contributes to a better understanding of the field and sets the stage for future innovations and research in dental implantology [154].

In the discussion of dental implant failures, it is imperative to delve into the dysfunctions of the immune system that can precipitate these complications. Implant failures are often associated with an overzealous immune response that can lead to chronic inflammation and peri-implantitis. For instance, an exaggerated immune reaction may result in the overproduction of pro-inflammatory cytokines such as TNF-α and IL-6, which not only exacerbate tissue damage but also disrupt the osseointegration process. Furthermore, an imbalance in the immune response can alter the oral microbiome, favoring the proliferation of pathogenic bacteria over commensal microflora, thereby increasing the risk of infection and implant rejection [155,156]. Additionally, autoimmune responses to implant materials can trigger hypersensitivity reactions, leading to implant instability and failure. Understanding these immune dysfunctions is pivotal in developing targeted therapies that can mitigate inflammatory responses and improve implant prognosis [38,90,157,158].

## 7. Conclusions

Dental implantology is a dynamically developing field of dentistry that addresses the problem of tooth loss, which reduces the quality of life of patients. Analyzing global statistics, it can be noticed that both clinicians and patients are increasingly interested in this method of treatment due to ensuring comfort of life and appropriate aesthetics. Although dental implantology is largely successful for most patients, it should be remembered that a complication such as periimplantitis is still a serious condition that has not been eliminated and may lead to the loss of the dental implant. That is why it is so important to understand the nature of the normal oral microflora and its impact on the immune system. An imbalance in the composition of microorganisms colonizing the oral cavity leads to disorders in the immune system, which might manifest itself in inflammation. Understanding how important it is to maintain oral hygiene is a breakthrough step that could reduce the occurrence or progression of many systemic diseases. Thanks to the development of research, the scientists could provide clinicians with more and relevant information on the influence of oral microflora on the successful osseointegration of implants. It is critical to constantly strive to develop new methods related to the modification of dental materials. Changes and improvements in these materials are also an important factor that could inhibit the colonization of pathogens on the surface of dental implants, which could reduce the incidence of periimplantitis.

## Data Availability

Not applicable.
